# Oxy210, a Semi-Synthetic Oxysterol, Inhibits Profibrotic Signaling in Cellular Models of Lung and Kidney Fibrosis

**DOI:** 10.3390/ph16010114

**Published:** 2023-01-12

**Authors:** Feng Wang, Frank Stappenbeck, Farhad Parhami

**Affiliations:** MAX BioPharma, Inc., 2870 Colorado Avenue, Santa Monica, CA 90404, USA

**Keywords:** oxysterols, fibrosis, TGF-β signaling, hedgehog (Hh) signaling

## Abstract

Oxy210, a semi-synthetic oxysterol derivative, displays cell-selective inhibition of Hedgehog (Hh) and transforming growth factor beta (TGF-β) signaling in epithelial cells, fibroblasts, and macrophages as well as antifibrotic and anti-inflammatory efficacy in models of liver fibrosis. In the present report, we examine the effects of Oxy210 in cellular models of lung and kidney fibrosis, such as human lung fibroblast cell lines IMR-90, derived from healthy lung tissue, and LL97A, derived from an idiopathic pulmonary fibrosis (IPF) patient. In addition, we examine the effects of Oxy210 in primary human renal fibroblasts, pericytes, mesangial cells, and renal tubular epithelial cells, known for their involvement in chronic kidney disease (CKD) and kidney fibrosis. We demonstrate in fibroblasts that the expression of several profibrotic TGF-β target genes, including fibronectin (*FN),* collagen 1A1 *(COL1A1),* and connective tissue growth factor (*CTGF)* are inhibited by Oxy210, both at the basal level and following TGF-β stimulation in a statistically significant manner. The inhibition of *COL1A1* gene expression translated directly to significantly reduced COL1A1 protein expression. In human primary small airway epithelial cells (HSAECs) and renal tubular epithelial cells, Oxy210 significantly inhibited TGF-β target gene expression associated with epithelial–mesenchymal transition (EMT). Oxy210 also inhibited the proliferation of fibroblasts, pericytes, and mesangial cells in a dose-dependent and statistically significant manner.

## 1. Introduction

Fibrosis, known as pathological scarring, can affect all tissues, including vital organs, such as kidney, liver, and lung, with deleterious consequences for organ function and human health. For fibrotic diseases in visceral organs, clinical outcomes (i.e., organ failure) remain dismal due to a paucity of effective treatment options that can slow or much less reverse the progression of fibrotic scarring. A causal nexus of tissue fibrosis lies in repeated micro-injuries to epithelial layers in the affected tissues that trigger non-resolving cycles of inflammation and pathological wound healing [1]. Among several different cell types (e.g., immune cells, fibroblasts, epithelial cells, endothelial cells, etc.) involved in these complex immune responses, activated myofibroblasts contribute significantly to irreversible scar formation.

Myofibroblasts are specialized fibrotic cells with contractile properties, characterized by overexpression of alpha-smooth muscle actin, chemotactic factors, and increased rates of cell proliferation [2]. Myofibroblasts are often derived from quiescent fibroblast progenitors in the connective tissues that migrate toward sites of injury when stimulated by various paracrine and autocrine factors [3]. However, in a profibrotic environment with repeated tissue injury, other cell types, such as resident epithelial cells or pericytes, may transdifferentiate and assume myofibroblast-like characteristics, expanding the pool of cells with fibrotic potential [4]. Activation and proliferation of myofibroblasts in pro-fibrotic lesions sets off cascading fibrotic events, such as over-production and accumulation of extracellular matrix (EM) components, including non-fibrillar collagens, hyaluronan, FN, and matricellular proteins, such as tenascins, thrombospondins, osteopontin and periostin [5]. Activation and proliferation of myofibroblasts and other cell types contributing to fibrosis are universally driven by profibrotic cellular signaling, i.e., growth factors, cytokines, and chemokines released in the local tissue microenvironment [6], a process that can even be further increased in the presence of EM and matricellular proteins, such as thrombospondin 1. These profibrotic signals include, most prominently, TGF-β signaling and several factors induced by TGF-β signaling, such as platelet-derived growth factor (PDGF) and CTGF [6]. In addition, contributions of other signaling pathways that are known for partial overlap and cross-talk with TGF-β signaling, including Hh, Notch, and Wnt signaling, have been reported [7,8,9,10]. Therefore, the selective inhibition of such profibrotic signals has been considered a therapeutic strategy for fibrotic conditions, such as non-alcoholic steatohepatitis (NASH), IPF, and renal fibrosis linked to CKD [11]. Paradoxically, TGF-β is a pleiotropic cytokine that, depending on the cellular context, can produce anti-inflammatory (i.e., immunosuppressive) or pro-inflammatory activities, in addition to the profibrotic properties mentioned above. Local release of TGF-β as a result of chronic injury or immune responses can have pro-inflammatory effects by stimulating the localized production of pro-inflammatory cytokines, such as interleukin-6 (IL-6) and tumor necrosis factor α (TNFα). TGF-β produced in vascular endothelial cells, for example, has been shown to advance pro-inflammatory activities and chronic vascular inflammation in atherosclerotic lesions [12]. With systemic release, the immunosuppressive properties of TGF-β often predominate, mediated largely through anti-inflammatory effects in monocytes and macrophages [13]. Hence, systemic and global inhibition of TGF-β signaling can be associated with pro-inflammatory side effects [14,15]. In addition, TGF-β signaling can stimulate proliferation in cells of mesenchymal origin, including myofibroblasts, whereas it tends to inhibit proliferation in epithelial, endothelial, and hematopoietic cells [13]. In epithelial cells, TGF-β is the major driver of epithelial–mesenchymal transition (EMT), along with minor contributions from other signaling pathways, such as Hh, Notch, and Wnt signaling [16]. The inappropriate activation of EMT, mediated by TGF-β signaling, has been implicated in the pathology of various forms of fibrosis [17,18], including cystic fibrosis ([19,20], and cancers that are particularly prone to early metastasis, such as pancreatic ductal adenocarcinoma [16].

Oxysterols are oxidized derivatives of cholesterol, known for wide-ranging biological activities that differ from those of cholesterol itself. Oxysterols, either naturally occurring or man-made, can be activators or inhibitors of cellular signaling. At MAX BioPharma, we strive to identify novel drug candidates among semi-synthetic oxysterol derivatives, following cycles of design, synthesis, and biological testing, an approach that we have termed Oxysterol Therapeutics^®^. During such studies, we discovered Oxy210, a synthetic oxysterol derivative, as a dual inhibitor of Hh and TGF-β signaling in cell cultures of A549 human lung epithelial cancer cells and NIH3T3 mouse fibroblast cells [21]. With respect to liver fibrosis, we characterized Oxy210 as an orally bioavailable drug candidate with antifibrotic properties, exhibited in vitro, in primary human hepatic stellate cells (HSCs), and in vivo, using the humanized APOE*3-Leiden.CETP mouse model of NASH [22]. In the NASH mouse model, oral administration of Oxy210 formulated in mouse food was well-tolerated over 16 weeks of continuous dosing and ameliorated several hallmarks of NASH, including hepatic inflammation, fibrosis, apoptosis, and lipid deposition in a dose-dependent manner, resulting in improved hepatic function ([22] and unpublished results). Given the potential for pro-inflammatory side effects associated with the systemic inhibition of TGF-β signaling, we were initially surprised to find that Oxy210 exerted anti-inflammatory effects in the liver, adipose tissue [23], and plasma of the mice, evidenced by reduced inflammatory cytokine expression and lowered cytokine levels in circulation [22]. In subsequent studies, we examined the effects of Oxy210 on macrophages in vitro and determined that Oxy210 can directly exert significant anti-inflammatory effects by inhibiting toll-like receptor (TLR) signaling in macrophages [23]. Notably, TGF-β signaling remains unaffected by Oxy210 in macrophages, confirmed by the absence of inhibitory effects on Smad2/3 phosphorylation, which preserves the anti-inflammatory effects of TGF-β ligands in these cells [23]. This cell-selective inhibition of TGF-β signaling by Oxy210 is new and different from other known modalities of TGF-β inhibition, such as small molecule TGF-β receptor 1 (TGFβRI/ALK5) inhibitors or TGF-β neutralizing antibodies, which tend to suppress TGF-β signaling across all cell types [21] with the potential for adverse events mentioned earlier. Encouraged by Hh and TGF-β inhibitory activity in HSCs and the combined antifibrotic and anti-inflammatory profile observed in APOE*3-Leiden.CETP mice [22,23], in the current study, we aim to demonstrate that Oxy210 can produce similar effects in cellular models of lung and kidney fibrosis driven by Hh and TGF-β signaling. Given additional favorable attributes of Oxy210 (oral bioavailability, drug-like pharmacokinetic and safety profiles, facile and scalable preparation, etc. [21]), we propose that Oxy210 and related molecules should be considered for future studies directed toward evaluation and therapeutic development as disease-modifying agents in IPF and CKD.

## 2. Results

### 2.1. Oxy210 Inhibits Mediators of Fibrosis in Cultures of Lung Fibroblasts

**Figure 1:**
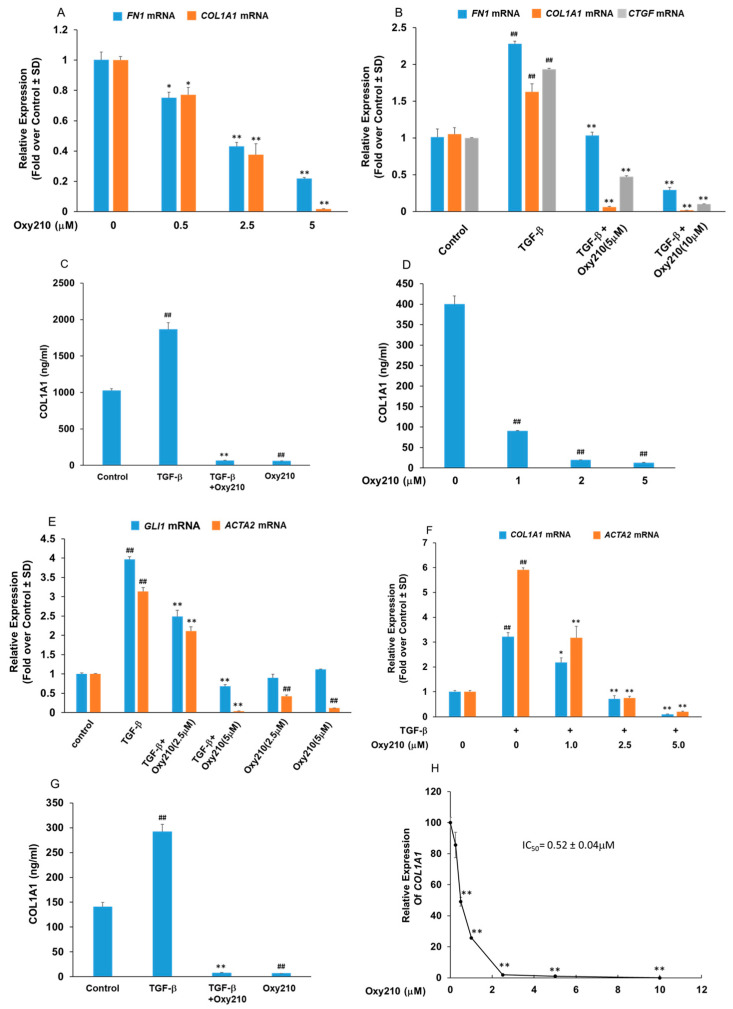
**Oxy210 inhibits the basal and TGF-β-induced expression of fibrotic genes**. (**A**) IMR-90 cells were treated with Oxy210 in DMEM containing 1% FBS for 72 h and then, RNA was analyzed by Q-RT-PCR for gene expression and normalized to *GAPDH*. Data from a representative experiment are reported as the mean of triplicate determinations ± SD (* *p* < 0.05 vs. control; ** *p* < 0.01 vs. control). (**B**,**E**) IMR-90 cells were pretreated with Oxy210 in DMEM containing 1% FBS for 24 h and then treated with TGF-β1 (10 ng/mL) in the absence or presence of Oxy210. After 48 h, RNA was analyzed by Q-RT-PCR for gene expression and normalized to *GAPDH*. Data from a representative experiment are reported as the mean of triplicate determinations ± SD (## *p* < 0.01 vs. control; ** *p* < 0.01 vs. TGF-β). (**C**) IMR-90 cells were pretreated with Oxy210 in DMEM containing 1% FBS for 24 h and then treated with TGF-β1 (10 ng/mL) in the absence or presence of Oxy210 (5 μM). After 72 h, the cells were lysed and the whole cell extracts were diluted and subjected to ELISA analysis for COL1A1. Data from a representative experiment are reported as the mean of triplicate determinations ± SD (## *p* < 0.01 vs. control; ** *p* < 0.01 vs. TGF-β). (**D**) IMR-90 cells were treated with Oxy210 in DMEM containing 1% FBS for 72 h. The cells were then lysed and the whole cell extracts were diluted and subjected to ELISA analysis for COL1A1. Data from a representative experiment are reported as the mean of triplicate determinations ± SD (## *p*< 0.01 vs. control). (**F**) LL97A cells were pretreated with Oxy210 in F12K medium containing 1% FBS for 24 h and then treated with TGF-β1 (10 ng/mL) in the absence or presence of Oxy210 as indicated. After 48 h, RNA was analyzed by Q-RT-PCR for gene expression and normalized to *GAPDH*. Data from a representative experiment are reported as the mean of triplicate determinations ± SD (## *p* < 0.01 vs. control; * *p* < 0.05 vs. TGF-β; ** *p* < 0.01 vs. TGF-β). (**G**) LL97A cells were pretreated with Oxy210 in F12K medium containing 1% FBS for 24 h and then treated with TGF-β1 (10 ng/mL) in the absence or presence of Oxy210 (5 μM). After 72 h, the cells were lysed and the whole cell extracts were diluted and subjected to ELISA analysis for COL1A1. Data from a representative experiment are reported as the mean of triplicate determinations ± SD (## *p* < 0.01 vs. control; ** *p* < 0.01 vs. TGF-β). (**H**) LL97A cells were treated with Oxy210 in F12K medium containing 1% FBS for 72 h. RNA was then analyzed by Q-RT-PCR for gene expression and normalized to *GAPDH*. Data from a representative experiment are reported as the mean of triplicate determinations ± SD (** *p* < 0.01 vs. control).

Fibrosis can be understood as an imbalance between EM deposition and degradation, including the unbalanced production and breakdown of extracellular collagen. Among various collagen subtypes, COL1A1 protein is the major component of type I collagen and by far the most abundant collagen present in human scar tissue. As such, COL1A1 can be considered a reliable biomarker in lung [24] and kidney [25] fibrosis and its reduction or removal in scar tissue may be therapeutically helpful. Lung myofibroblasts, stimulated by TGF-β and other profibrotic factors, are considered central mediators of the pathological fibrotic EM accumulation, including collagen, FN, and CTGF, all TGF-β target genes known for their pivotal roles in fibrosis [26]. We employed the IMR-90 cells, a human lung fibroblast cell line derived from healthy fetal lung tissue, to study the effect of Oxy210 on the expression of pro-fibrotic genes. As shown in Figure 1A,B, Oxy210 treatment inhibited the baseline expression of *FN1* and *COL1A1* as well as the TGF-β-induced expression of *FN1*, *COL1A1*, and *CTGF*, respectively, in a dose-dependent and statistically significant manner. Among the genes stimulated by TGF-β, the expression of *COL1A1* appears to be more sensitive to inhibition by Oxy210 compared to *FN1* and *CTGF* (Figure 1A,B). The effect of Oxy210 on cellular COL1A1 protein expression was also examined using ELISA. As shown in Figure 1C, treatment of IMR-90 cells with 5 μM Oxy210 resulted in a significant decrease in basal and TGF-β stimulated COL1A1 protein expression (measured by ELISA in cell lysate). The reduction in basal COL1A1 protein levels by Oxy210 was dose-dependent showing a 4-fold reduction at 1 μM and a 40-fold reduction at 2 and 5 μM (Figure 1D). These data suggest that Oxy210 can effectively suppress COL1A1 protein expression in IMR-90 cells in a dose-dependent and statistically significant manner. It has been reported that Hh signaling alone can stimulate myofibroblast differentiation and the release of collagen in vitro and is sufficient to induce fibrosis in vivo, according to published reports [27]. Non-canonical Hh signaling can be stimulated by TGF-β [28] and myofibroblast differentiation is marked by the induced expression of α-smooth muscle actin (*ACTA2*) [29]. As shown in Figure 1E, Oxy210 inhibited the TGF-β-induced expression of *GLI1* and *ACTA2* in a dose-dependent and statistically significant manner in IMR-90 cells. While the expression of *ACTA2* was significantly reduced at the basal level and when induced by the TGF-β ligand, the expression of *GLI1* was inhibited by Oxy210 only when stimulated with the TGF-β ligand. The latter result is consistent with the absence of significant baseline *GLI1* expression and Hh signaling which is stimulated by TGF-β. The effect of Oxy210 on pro-fibrotic gene expression was also studied in LL97A cells, an IPF patient-derived human lung fibroblast cell line. Oxy210 significantly inhibited the basal and TGF-β stimulated expression of *COL1A1* and *ACTA2* (Figure 1F) and COL1A1 protein expression (Figure 1G) to below basal levels. In addition, Oxy210 significantly inhibited basal COL1A1 protein expression in LL97A cells with a half maximal inhibitory concentration (IC_50_) of 0.52 ± 0.04 μM (Figure 1H). Unsurprisingly, these results are consistent with a tight correlation between the COL1A1 gene and protein expression, at least in two relevant human lung fibroblast cell lines, IMR-90 and LL97A. In future studies, we plan to provide further confirmation in human primary cells.

### 2.2. Profibrotic Gene Expression in Lung Fibroblasts Is Regulated by Hh and TGF-β Signaling

**Figure 2:**
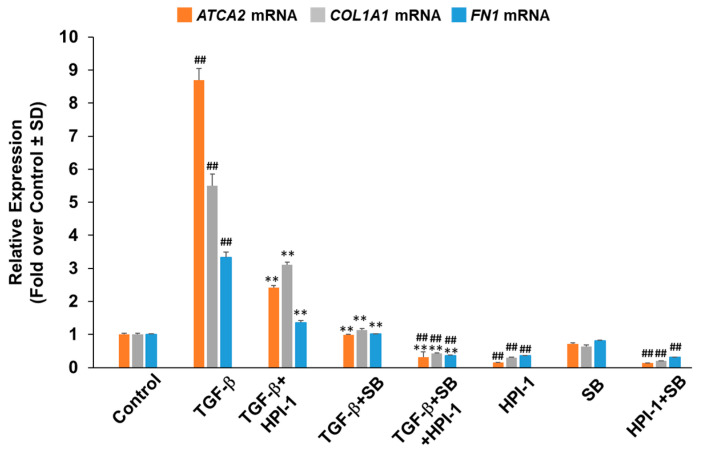
**HPI-1 and SB-431542, known inhibitors of Hh and TGF-β signaling, reduce the basal and TGF-β-induced expression of fibrotic genes**. LL97A cells were treated with TGF-β1 (10 ng/mL) in F12K medium containing 1% FBS in the absence or presence of HPI-1 (5 μM) or SB-431542 (SB) (5 μM) as indicated. After 48 h, RNA was analyzed by Q-RT-PCR for gene expression and normalized to GAPDH. Data from a representative experiment are reported as the mean of triplicate determinations ± SD (## *p* < 0.01 vs. control; ** *p* < 0.01 vs. TGF-β).

We have previously hypothesized that Oxy210 through simultaneous inhibition of both Hh and TGF-β signaling pathways may be therapeutically more effective compared to selective inhibitors of the Hh or TGF-β pathways alone [21,22]. To separate individual contributions of Hh and TGF-β signaling to responses in profibrotic gene expression, LL97A cells were studied in the presence or absence of TGF-β induction, and treated with HPI-1, a selective inhibitor of Gli transcription factors [30] and/or SB-431542 (SB), a selective TGFβRI/ALK5 inhibitor [31]. As shown in Figure 2, expression of *ACTA2*, *COL1A1*, and *FN*, was significantly suppressed by either HPI-1 or SB, with or without TGF-β stimulation, suggesting that profibrotic responses may be partially regulated by both signaling pathways. Combination treatments of HPI-1 and SB, with or without TGF-β stimulation, suppressed profibrotic gene expression below basal levels, hinting at possible synergy in this inhibition between Hh/Gli and TGF-β signaling. HPI-1 and SB treatment alone, in the presence of TGF-β stimulation, did not reduce profibrotic gene expression below baseline. Hence, the effect of Oxy210 on profibrotic gene expression in LL97A cells may resemble the combination treatment of HPI-1 and SB.

### 2.3. Oxy210 Inhibits Proliferation of Pulmonary Fibroblasts

**Figure 3:**
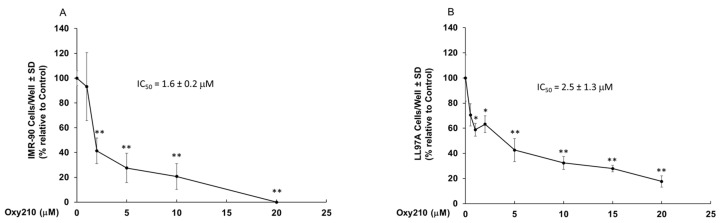
**Oxy210 inhibits proliferation of IMR-90 and LL97A cells.** IMR-90 (**A**) or LL97A (**B**) were treated with Oxy210 as indicated in DMEM or F12K containing 5% FBS, respectively, for 6 days and then were trypsinized and counted. Data from a representative experiment are reported as the mean of triplicate determinations ± SD (* *p* < 0.05 vs. control; ** *p* < 0.01 vs. control).

Fibroblasts proliferation and differentiation occur in response to prolonged tissue injury as well as chronic inflammation and activated lung fibroblasts are characterized by enhanced proliferation [32]. To examine the effect of Oxy210 on the proliferation of pulmonary fibroblasts in vitro, cell counting experiments were conducted in the presence of increasing concentrations of Oxy210 using the IMR-90 and LL97A cells. Treatment of cells with Oxy210 resulted in the inhibition of proliferation with an IC_50_ of 1.6 ± 0.17 μM for IMR-90 cells and 2.5 ± 1.3 μM for LL97A cells (Figure 1B and Figure 3A). It is noteworthy that culturing of various fibroblastic and non-fibroblastic cells, including pulmonary fibroblasts, HSCs, and pericytes, on plastic tissue culture plates can result in an activated state and enhanced proliferative activity [33,34,35]. These states are inhibited by Oxy210, evidenced in this report by the inhibition of baseline expression of activation markers *ACTA2* and *COL1A1* in both lung fibroblasts employed in our studies (Figure 1E,G).

### 2.4. Oxy210 Inhibits Expression of Epithelial–Mesenchymal Transition (EMT) Genes and Interleukin-6 in Human Primary Small Airway Epithelial (HSAE) Cells

**Figure 4:**
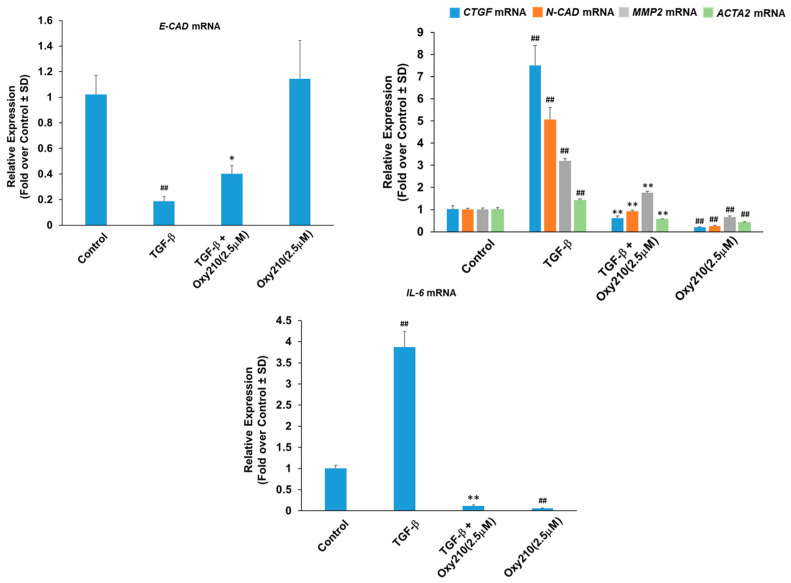
**Oxy210 inhibits the expression of TGF-β target genes in HSAE cells including EMT genes and the inflammatory cytokine, *IL-6***. HSAE cells were pretreated with Oxy210 in DMEM containing 1% FBS for 4 h and then treated with TGF-β1 (10 ng/mL) in the absence or presence of Oxy210. After 72 h, RNA was analyzed by Q-RT-PCR for gene expression and normalized to *GAPDH*. Data from a representative experiment are reported as the mean of triplicate determinations ± SD (## *p* < 0.01 vs. control; * *p* < 0.05 vs. TGF-β; ** *p* < 0.01 vs. TGF-β).

Repetitive injuries to the alveolar epithelium as well as inappropriate responses of airway epithelial cells to such injuries may contribute significantly to disease development and progression in IPF. This suggests that airway epithelial cells, such as small airway epithelial (HSAE) cells, could add fibrogenic potential through EMT stimulated via profibrotic signaling (e.g., TGF-β, Hh, Notch, and Wnt signaling) or hypoxia [36,37,38]. EMT is a patho-physiological process through which, in various diseases, epithelial cells acquire the phenotype of mesenchymal cells and express EM molecules that contribute to fibrosis [39,40]. EMT is orchestrated on the transcriptional level by the upregulation of a network of transcription factors, including SNAIL and TWIST, that directly repress epithelial genes and upregulate mesenchymal gene markers [41]. The source of myofibroblasts contributing to IPF is not completely understood. However, pulmonary epithelial cells undergoing EMT can reportedly play a role in creating a profibrotic environment in the lung even if they themselves do not account for a significant source of myofibroblasts in IPF [42]. Oxy210 significantly inhibited TGF-β-stimulated expression of the mesenchymal markers *CTGF*, matrix metalloproteinase 2 (*MMP2*), *ACTA2*, and N-cadherin (*N-CAD*) (Figure 4) and partially reversed the TGF-β-induced reduction in the epithelial marker E-cadherin (*E-CAD*) (Figure 4). In addition, TGF-β-induced the expression of *IL-6* by HSAE cells, an effect that was inhibited to below basal levels by Oxy210 (Figure 4). It is noteworthy that IL-6 has been shown to have pleiotropic effects that can be antifibrotic or pro-fibrotic, and pro-inflammatory in bleomycin-induced lung fibrosis in mice [37,43].

### 2.5. Oxy210 Inhibits Mediators of Fibrosis in Cultures of Kidney Cells

**Figure 5:**
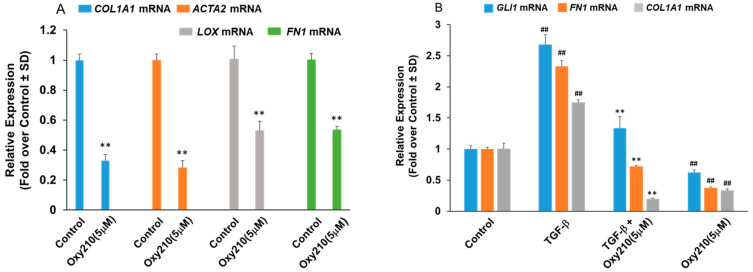
**Effect of Oxy210 on basal and TGF-β-induced expression of fibrotic genes in primary human pericytes.** (**A**) Human pericytes were treated with control vehicle or Oxy210 for 48 h in DMEM containing 1% FBS. RNA was extracted and analyzed by Q-RT-PCR for the expression of pro-fibrotic genes and normalized to *GAPDH* expression. Data from a representative experiment are reported as the mean of triplicate determinations ± SD (** *p* < 0.01 vs. control). (**B**) Human pericytes cultured in DMEM containing 1% FBS were treated with TGF-β1 (10 ng/mL) in the absence or presence of Oxy210 for 48 h. RNA was extracted and analyzed by Q-RT-PCR for the expression of pro-fibrotic genes and normalized to *GAPDH* expression. Data from a representative experiment are reported as the mean of triplicate determinations ± SD (## *p* < 0.01 vs. control; ** *p* < 0.01 vs. TGF-β).

**Figure 6:**
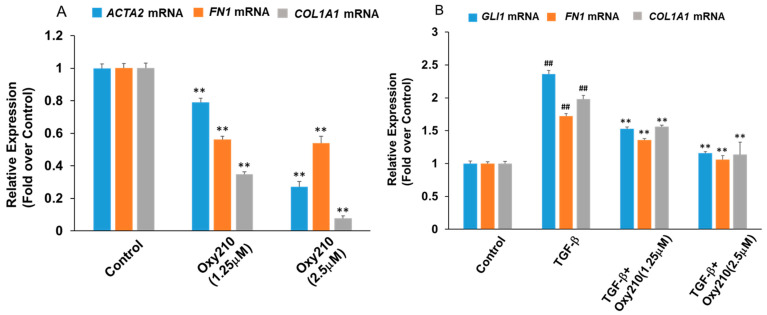
**Inhibition of basal and TGF-β-induced expression of fibrotic genes in human renal fibroblast cells by Oxy210.** (**A**) human renal fibroblast cells were treated with Oxy210 in DMEM containing 1% FBS for 48 h and then RNA was extracted and analyzed by Q-RT-PCR for the expression of the genes and normalized to *GAPDH* expression. Data from a representative experiment are reported as the mean of triplicate determinations ± SD (** *p* < 0.01 vs. control). (**B**) Human renal fibroblast cells were pretreated with Oxy210 in DMEM containing 1% FBS for 2 h and then treated with TGF-β1 (10 ng/mL) in the absence or presence of Oxy210 for 48 h. RNA was extracted and analyzed by Q-RT-PCR for the expression of the genes and normalized to *GAPDH* expression. Data from a representative experiment are reported as the mean of triplicate determinations ± SD (## *p* < 0.01 vs. control; ** *p* < 0.01 vs. TGF-β).

Progressive tissue fibrosis is known as a common underlying mechanism of chronic kidney conditions, preceding end-stage kidney disease and organ failure. To demonstrate the potential therapeutic utility of Oxy210 in this context, we examined various primary human kidney cell types implemented in chronic kidney disease. Renal pericytes, ubiquitous perivascular cells, have attracted interest in kidney fibrosis as they can be potentially recruited as interstitial myofibroblast precursors and have been reported to play critical roles in angiogenesis and regulation of renal fibrosis [44]. As shown in Figure 5A, Oxy210 treatment at 5 μM resulted in a significant reduction in the expression of four prominent profibrotic genes, *COL1A1*, *ACTA2*, protein-lysine 6-oxidase (*LOX*), and *FN1* in primary human pericytes (Figure 5A). TGF-β stimulation of these cells resulted in upregulated expression of *COL1A1*, *FN1*, and *GLI1*, and TGF-β-induced upregulation of these genes was significantly inhibited by Oxy210 (Figure 5B). Moreover, renal fibroblasts are believed to be the effector cells in renal fibrosis and are in part responsible for the synthesis and deposition of EM components [45]. Oxy210 treatment alone reduced the expression of *ACTA2*, *FN1*, and *COL1A1* in human primary renal fibroblasts (Figure 6A). TGF-β stimulation of these cells produced a modest but significant increase in *ACTA2*, *FN1*, and *COL1A1* expression which was reduced to near basal levels by Oxy210 (Figure 6B). Under these experimental conditions, the profibrotic genes have a high basal expression.

### 2.6. Oxy210 Inhibits Proliferation of Primary Human Pericytes, Renal Fibroblasts and Renal Mesangial Cells

**Figure 7:**
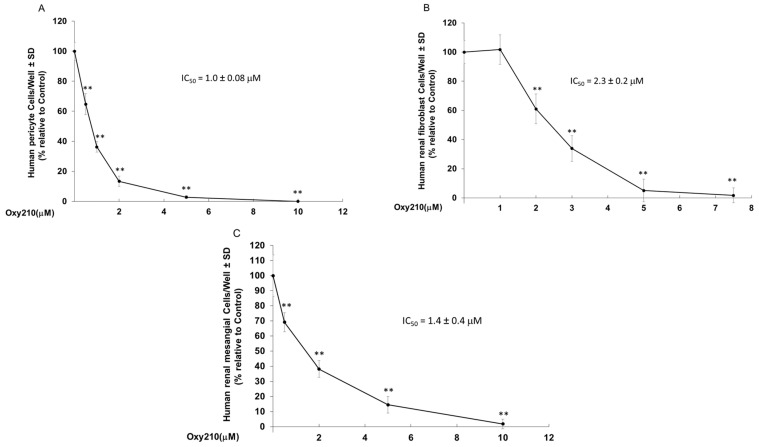
**Oxy210 inhibits proliferation of human pericytes, renal fibroblasts, and renal mesangial cells**. Human pericytes (**A**), human renal fibroblast cells (**B**) or human renal mesangial cells (**C**) were treated with Oxy210 as indicated in DMEM or RPMI containing 5% FBS, respectively, for 6 days and then were trypsinized and counted. Data from a representative experiment are reported as the mean of triplicate determinations ± SD (** *p* < 0.01 vs. control).

Activated renal pericytes and fibroblasts are characterized by enhanced proliferative states and aberrant proliferation of renal mesangial cells is a common finding in several kidney diseases that can lead to end-stage renal failure [46]. To examine the effect of Oxy210 on the proliferation of these cells, primary human pericytes, renal fibroblasts, and mesangial cells were cultured, and cell counting experiments were performed in the presence of increasing concentrations of Oxy210. Oxy210 inhibited the proliferation of all cell types with IC50s of 1.0 ± 0.08 μM for pericytes (Figure 7A), 2.3 ± 0.17 μM for renal fibroblasts (Figure 7B), and 1.4 ± 0.4 μM for renal mesangial cells (Figure 7C).

### 2.7. Oxy210 Inhibits TGF-β-Induced EMT Gene Expression of Human Primary Renal Tubular Epithelial Cells

**Figure 8:**
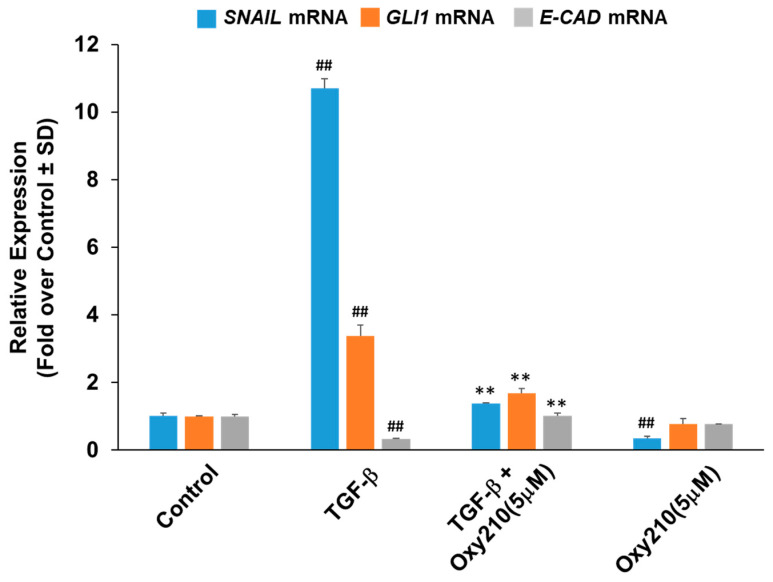
**Inhibition of TGF-β-induced EMT gene expression in renal tubular epithelial cells by Oxy210.** Human renal proximal tubular epithelial cells cultured in RPMI containing 1% FBS were treated with TGF-β1 (10 ng/mL) in the absence or presence of Oxy210 for 48 h. RNA was extracted and analyzed by Q-RT-PCR for the expression of the genes as indicated and normalized to *GAPDH* expression. Data from a representative experiment are reported as the mean of triplicate determinations ± SD (## *p* < 0.01 vs. control; ** *p* < 0.01 vs. TGF-β).

Complete EMT or partial EMT (pEMT) of renal tubular epithelial cells, induced by Hh, TGF-β, and other signaling, is regarded as one of several mechanisms that promote renal fibrosis [47]. During EMT or pEMT, injured epithelial cells are activated and undergo a phenotypic conversion to acquire some features of matrix-producing myofibroblasts [48]. When stimulated by TGF-β, human primary renal tubular epithelial cells exhibited significant increases in the expression of *SNAIL*, a key EMT regulator, and *GLI1*, a Hh target gene, as well as a decrease in E-cadherin (*E-CAD*) expression, an epithelial marker [49], and treatment with Oxy210 reversed these TGF-β-induced responses (Figure 8).

## 3. Discussion

A range of fatal human diseases are directly associated with fibrosis, such as end-stage liver disease (cirrhosis), end-stage CKD, IPF, and heart failure. In addition, metabolic conditions, such as diabetes and obesity, and some autoimmune diseases, such as rheumatoid arthritis, Crohn’s disease, and scleroderma, can be accompanied by fibrotic tissue remodeling [11]. Pathological scarring is also observed in many cancers and may enhance tumor invasion and metastasis [50]. Fibrotic conditions are universally driven by aberrant activation of profibrotic signaling, often in combination with non-resolving inflammation. Among profibrotic signals, Hh and TGF-β are not only involved in the progression of fibrotic diseases involving liver, lung, and kidney [51,52,53] but they can also play key roles in the development and progression of cancer, such as lung, renal, and liver cancer [54,55,56,57,58,59]. Non-small cell lung cancer (NSCLC), for example, stands out among comorbidities diagnosed in IPF patients [60,61]. Furthermore, pirfenidone, a drug used in the treatment of IPF, which may also provide clinical benefits in renal and liver fibrosis [62,63,64], is believed to attain some of its benefits through modulation of both the Hh and TGF-β signaling pathways [65]. Pirfenidone has been demonstrated to interact with the transcription factor GLI2, a point of convergence between the Hh and TGF-β signaling pathways, and promote GLI2 degradation, albeit with modest potency [65]. Nintedanib, the only other marketed drug for IPF, is known to inhibit early events in TGF-β signaling, specifically the phosphorylation of TGF-β receptor 2 (TGFBR2) and Smad3 activation [66]. Given the relevance of Hh and TGF-β signaling as drivers of fibrosis and associated cancers, we propose that the effective inhibition of these pathogenic cellular signaling pathways could potentially yield improved efficacy and eventual clinical benefits, compared to the existing therapies, such as pirfenidone and nintedanib. Our data presented in the present report are consistent with the potential of a dual inhibitor of Hh and TGF-β signaling, for example, Oxy210, as a therapy for lung fibrosis and perhaps other fibrotic diseases. Liver, lung, and kidney fibrosis share some common characteristics: Repeated injuries to the epithelia are believed to activate myofibroblast differentiation, proliferation, and migration, creating a profibrotic environment that prevents productive wound healing. In the liver, the activation of HSCs, derived from a small population of quiescent, vitamin A-storing liver cells (localized between hepatic endothelial and sinusoidal spaces), into proliferative and fibrotic myofibroblasts constitutes a central driver of liver fibrosis, including common forms of NASH [67]. IPF is a chronic interstitial lung disease characterized by progressive, irreversible scarring of lung tissue and declining lung function, terminating in respiratory failure [68,69]. IPF affects both structural cells, such as lung epithelial cells and fibroblasts, as well as immune cells, such as macrophages. Activated lung myofibroblasts are known to advance and aggravate IPF. CKD, in its various forms, converges on renal fibrosis as a final common pathway leading to end-stage kidney disease, i.e., renal failure and death. Renal myofibroblasts are known to exacerbate kidney fibrosis, a complex and irreversible condition that may also involve other renal cell types, such as renal pericytes, mesangial cells, and renal tubular epithelial cells. These cells can be activated and may proliferate aberrantly or undergo phenotypic conversions to acquire partial features of EM-producing myofibroblasts [44,45,47,70,71]. The patho-physiological process by which epithelial cells lose part of their characteristics and markers, while gaining mesenchymal properties, including a tendency to overproduce EM components, is known as EMT and is most often mediated by TGF-β signaling with possible contributions from other profibrotic signaling (e.g., Hh, Notch and Wnt signaling) or hypoxia [16,36,37,38,39,40,41,42]. EMT, or partial EMT, is a known clinical feature in several liver, lung, and kidney diseases, including NASH [72], NSCLC [21,73], and kidney fibrosis [71], and a role for EMT in the pathogenesis of IPF and as a possible source of myofibroblasts has been hypothesized [42]. Short of being a major source of myofibroblasts in IPF, alveolar epithelial cells undergoing EMT may still promote a pro-fibrotic microenvironment through paracrine signaling which activates local fibroblasts, according to published reports [42]. Here, we demonstrate that Oxy210 effectively suppresses the expression of TGF-β modulated EMT target genes in primary lung and renal epithelial cells.

In earlier reports, we described the beneficial effects of Oxy210, a dual inhibitor of Hh and TGF-β signaling, on profibrotic responses observed in HSCs in vitro, and on the progression of liver fibrosis in vivo, using a humanized mouse model of NASH [22]. We also reported considerable anti-inflammatory effects of Oxy210 in vitro in macrophages and in vivo, in the liver, adipose tissue [23], and plasma of mice [22]. In the present follow-up report, we investigate the effects of Oxy210 in cellular models of lung and kidney fibrosis. Our data demonstrate that Oxy210 reliably inhibits proliferation, pro-fibrotic responses, and EMT gene expression in lung and kidney cell types that are known mediators of pulmonary and kidney fibrosis, such as in lung fibroblast cells lines, primary HSAE cells as well as primary human renal fibroblasts, pericytes, mesangial cells, and renal tubular epithelial cells. These data are consistent with the previously reported antifibrotic effects of Oxy210 in HSCs [22] as well as its anti-proliferative and inhibitory effects on EMT gene expression reported in A549 NSCLC cells [21]. These in vitro effects of Oxy210 are observed at concentrations in the low micromolar range, at or below Oxy210 plasma levels and levels of Oxy210 detected in liver and lung tissue following oral administration of Oxy210 in mice [74]. Significant therapeutic effects may therefore be achievable with Oxy210 in mouse models of lung and kidney fibrosis. Conceptually, TGF-β signaling has long been recognized as an attractive therapeutic target for intervention in cancer and fibrosis, including liver [51], pulmonary [52,55,73,75,76], and kidney fibrosis [53,57,77]. However, as mentioned earlier, the direct, global, and complete inhibition of TGF-β signals has been linked to pro-inflammatory side effects, including cardiac valve abnormalities as well as vascular and renal inflammation [14,15]. TGF-β knockout mice develop systemic autoimmune disorders, are more susceptible to cancer, and die of massive inflammation [78,79]. Several clinical trials of systemic TGF-β antibody treatments had to be terminated because of dose-limiting adverse events and the clinical development of other TGF-β inhibitors, such as small molecule TGFβRI/ALK5 antagonists, has been slow to progress for similar reasons. To address the narrow therapeutic window of these TGF-β inhibitors, mitigation strategies had to be developed that include co-administration with anti-inflammatory therapies or dose duration limits. Similar strategies include indirect or site-specific TGF-β inhibition, for example, by blocking integrins, latency proteins, and other local mediators of TGF-β signaling [78]. Alternatively, cell-selective modalities of TGF-β inhibition could potentially overcome many of these obstacles through specific targeting of activated myofibroblasts that avoids interfering with the anti-inflammatory effects of TGF-β in leukocytes. In pioneering proof of principle studies, the fibroblast-specific inhibition of TGF-β signaling mediated by naturally occurring polyphenols, such as ellagic acid and corilagin, has been reported to attenuate lung and tumor fibrosis [80]. In this regard, our previous findings revealed the remarkable ability of Oxy210 to inhibit TGF-β signaling in fibroblasts without disrupting the anti-inflammatory effects of this pleiotropic cytokine in macrophages [23]. We have identified the inhibition of toll-like receptor (TLR) 2 and 4 signaling by Oxy210 as a mechanistic origin of these anti-inflammatory effects [23]. TLR signaling pathways are often activated in chronic inflammatory diseases [81] and are also tied to many fibrotic conditions, including NASH [82,83], IPF [84], and CKD [85]. By inhibiting cellular responses of both chronic inflammation and the profibrotic signals, the use of oxysterol-based inhibitors, such as Oxy210, could potentially achieve both efficacy and improved drug safety associated with TGF-β inhibition. All clinically approved Hh pathway inhibitors to date (vismodegib, sonidegib, and glasdegib) are inhibitors of the G protein-like coupled receptor Smoothened (Smo), so-called Smo antagonists, and used primarily in the treatment of basal cell carcinoma and acute myeloid leukemia [86]. Since activation of Hh signaling has been reported in IPF, attempts have been made to examine one of these FDA-approved drugs, vismodegib, for the treatment of pulmonary fibrosis in a phase 1b clinical study, administered in combination with pirfenidone. Unfortunately, drug safety issues of vismodegib emerged as a limiting factor for treating IPF during the study [87]. Based on a different mechanism of Hh pathway inhibition, we propose that Oxy210 could potentially be a safer and more effective compound for applications in fibrosis compared to vismodegib. Unlike vismodegib, Oxy210 is not a Smo antagonist and inhibits Hh signaling at the level of Gli activity [21]; hence, allowing for non-Gli mediated effects of Smo to remain intact [88]. In addition, preliminary reproductive toxicology studies in CD1 mice indicate that Oxy210 appears to be devoid of teratogenic properties that have been documented for the entire class of Smo antagonists, including vismodegib [22,89]. Inhibition of Hh signaling has also been deemed an attractive strategy in targeting kidney fibrosis as well [90,91,92,93], although no clinical trials of Smo antagonists have been reported in the context of CKD.

In summary, unique features of Oxy210 include its ability to antagonize TGF-β and Hh signaling in fibroblasts, EMT gene expression in epithelial cells as well as cytokine signaling that contributes to macrophage activation and chronic inflammation [21,22,23].

The results of the current study demonstrate that an array of relevant lung and kidney cell types, such as lung fibroblast cell lines, primary HSAE cells, as well as primary human renal fibroblasts, pericytes, mesangial cells, and renal tubular epithelial cells, all respond favorably to Oxy210 treatment. However, limitations of our study include: (1) The findings described in IMR-90 and LL97A cell lines need to be confirmed in primary human lung fibroblasts; (2) the efficacy of Oxy210 will have to be confirmed and further evaluated in animal models of lung and kidney fibrosis; and (3) ongoing drug safety studies will further evaluate Oxy210 as a drug candidate suitable for clinical development.

## 4. Materials and Methods

### 4.1. Cell Culture and Reagents

**Figure 9:**
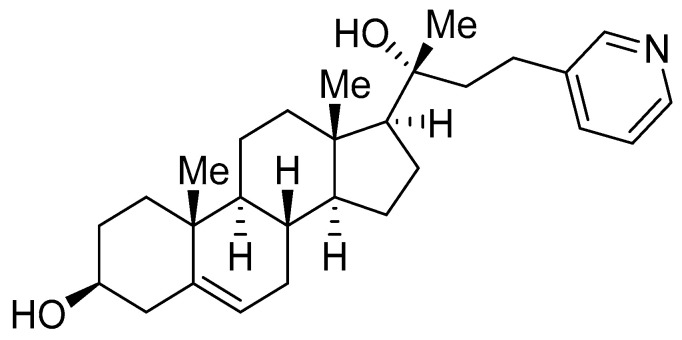
Molecular formula of Oxy210.

IMR-90, LL97A, human pericyte, human renal fibroblast cells, and human renal mesangial cells were obtained from ATCC (Manassas, VA, USA). IMR-90, human renal fibroblast cells, and human pericytes were cultured in DMEM containing 10% FBS. LL97A and human renal mesangial cells were cultured in F12K and RPMI containing 10% FBS, respectively. TGF-β1 was obtained from R&D Systems (Minneapolis, MN, USA). Oxy210 (Figure 9) was prepared by MAX BioPharma, according to a previously reported procedure [21].

### 4.2. Quantitative RT-PCR

Total RNA was extracted with the RNeasy Plus Mini Kit from Qiagen (Hilden, Germany) according to the manufacturer’s instructions. One microgram of RNA was reverse-transcribed using an iScript Reverse Transcription Supermix from Bio-Rad Laboratories (Hercules, CA, USA) to make single-stranded cDNA. The cDNAs were then mixed with Qi SYBR Green Supermix (Bio-Rad) for quantitative RT-PCR assay using a Bio-Rad I-cycler IQ quantitative thermocycler. All PCR samples were prepared in triplicate wells in a 96-well plate. After 40 cycles of PCR, melt curves were examined in order to ensure primer specificity. Fold changes in gene expression were calculated using the ΔΔCt method. Primers used for mouse were as follows: Oaz1 (5′-CCACTGCTTCGCCAGAGAG-3′) and (5′-CCCGGACCCAGGTTACTA-3′); Gli1 (5′- GCTTGGATGAAGGACCTTGTG-3′ and 5′-GCT GATCCAGCCTAAGGTTCTC-3′); CTGF (5′-GGGCCTCTTCTGCGATTTC-3′ and 5′-ATCCAGGCAAGTGCATTGGTA-3′). Primers used for human were as follows: GAPDH (5′-CCTCAAGATCATCAGCAATGCCTCCT-3′ and 5′-GGTCATGAGTCCTTCCACGATACCAA-3′); CTGF (5′-CAGCATGGACGTTCGTCTG-3′) and (5′-AACCACGGTTTGGTCCTTGG-3′); GLI1 (5′-GAAGCCGAGCCGAGTATC-3′ and 5′-CGGTGGTTTCTT GGTCGGT-3′); COL1A1 (5′-GTGCGATGACGTGATCTGTGA-3′ and 5′-ACTCACTGCTCTGCTTGTTCTG-3′); Fn-EDA (5′-AGGAAGCCGAGGTTTTAACTG-3′ and 5′-AGGACGCTCATAAGTGTCACC-3′); E-cadherin (5′-ATTTTTCCCTCGACACCCGAT-3′ and 5′-TCCCAGGCGTAGACCAAGA-3′); SNAIL (5′-TCGGAAGCCTAACTACAGCGA-3′ and 5′-AGATGAGCATTGGCAGCGAG-3′); ACTA2 (5′-GTGTTGCCCCTGAAGAGCAT-3′ and 5′-GCTGGG ACATTGAAAGTCTCA-3′); LOX (5′-ACCACAGGCGATTTGCATGTA-3′ and 5′-GGCAGTCTATGT CTGCACCA-3′).

### 4.3. ELISA Assay

The human Pro-Collagen I alpha 1 SimpleStep ELISA kit was purchased from Abcam (Cambridge, UK). ELISA was performed according to the manufacturer’s instructions. Briefly, the cells were cultured in 6-well plates and treated with the test reagents for 72 h and then were lysed in 200 μL cell extraction buffer PTR. Then, the cell extracts were suitably diluted with PTR and subjected to the assay together with the serial diluted ProCollagen I alpha protein. The protein concentration was measured using Bio-Rad Protein Assay dye reagent (Bio-Rad, Hercules, CA, USA). The OD was recorded at 450 nM.

### 4.4. Cell Counting Assay

IMR-90 and human pericytes cultured in DMEM containing 5% FBS, and LL97A and human renal mesangial cells cultured in F12K and RPMI, respectively, containing 5% FBS in 12-well plates at 20% confluence were treated with Oxy210 for 6 days and then trypsinized, spun down and resuspended in fresh medium. An aliquot of cell suspension was applied to a hemocytometer and the cells were then counted under a light microscope.

### 4.5. Statistical Analysis

Statistical analyses were performed using the StatView 5 program (SAS Institute, Cary, NC, USA). All *p*-values were calculated using ANOVA and Fisher’s projected least significant difference (PLSD) significance test. A value of *p* < 0.05 was considered significant. The IC_50_ dose–response curves were modeled using a five-parameter logistic model. This model allows for asymmetric curves and automatically estimates the mean maximum and minimum response. Based on this model, IC_50_ values were estimated corresponding to the dose halfway between the min and max response. Models of dose versus response and dose versus log response were also evaluated. The R square statistic was computed as a measure of model fit.

## 5. Conclusions

In this report, we have demonstrated the potential utility of Oxy210 as an antifibrotic drug candidate in cellular models of myofibroblasts activation and proliferation using lung and kidney cells. Based on the antifibrotic profile of Oxy210 observed in these models along with previously reported antifibrotic and anti-inflammatory effects in mice, we conclude that Oxy210 and related oxysterols should be further evaluated as potential disease-modifying agents in the treatment of fibrotic conditions, including NASH, IPF, and kidney fibrosis. As the apparent targets of Oxy210 are key cellular and molecular drivers of human organ fibrosis and given the fact that tissue fibrosis often involves chronic inflammation in humans, eventual clinical testing could determine whether Oxy210 or related oxysterols can help preserve or improve organ function in patients suffering from liver, lung, and kidney fibrosis.

## Data Availability

The data presented in this study are available on request and within reason from the corresponding author (F.P.). The data are not publicly available due to privacy.
